# The genome sequence of a leaf beetle, *Cryptocephalus moraei *(Linnaeus, 1758)

**DOI:** 10.12688/wellcomeopenres.19522.1

**Published:** 2023-10-13

**Authors:** Olga Sivell, Duncan Sivell, Ryan Mitchell, Natural History Museum Genome Acquisition Lab, Darwin Tree of Life Barcoding collective, Wellcome Sanger Institute Tree of Life programme, Wellcome Sanger Institute Scientific Operations: DNA Pipelines collective, Tree of Life Core Informatics collective, Darwin Tree of Life Consortium

**Affiliations:** 1 Natural History Museum, London, England, UK

**Keywords:** Cryptocephalus moraei, a leaf beetle, genome sequence, chromosomal, Coleoptera

## Abstract

We present a genome assembly from an individual female *Cryptocephalus moraei* (a leaf beetle; Arthropoda; Insecta; Coleoptera; Chrysomelidae). The genome sequence is 500.5 megabases in span. Most of the assembly is scaffolded into 15 chromosomal pseudomolecules, including the X sex chromosome. The mitochondrial genome has also been assembled and is 15.68 kilobases in length.

## Species taxonomy

Eukaryota; Metazoa; Eumetazoa; Bilateria; Protostomia; Ecdysozoa; Panarthropoda; Arthropoda; Mandibulata; Pancrustacea; Hexapoda; Insecta; Dicondylia; Pterygota; Neoptera; Endopterygota; Coleoptera; Polyphaga; Cucujiformia; Chrysomeloidea; Chrysomelidae; Cryptocephalinae; *Cryptocephalus*; *Cryptocephalus moraei* (Linnaeus, 1758) (NCBI:txid204949).

## Background

**Figure 1.**
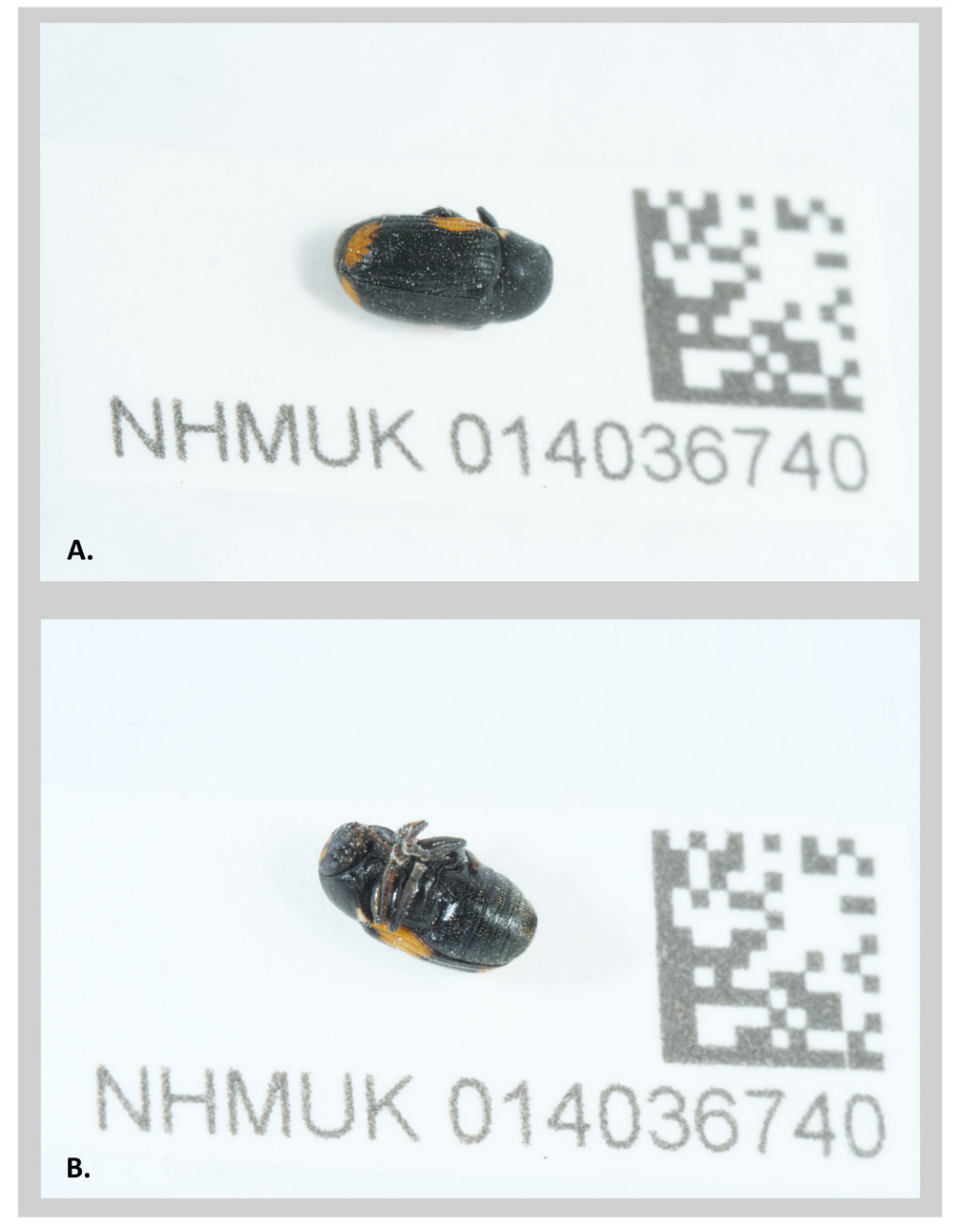
Photographs of the *Cryptocephalus moraei* specimen NHMUK014036740 (icCryMora2) taken during sample preservation and processing. **A**. Habitus of the specimen in dorsal view. **B**. The specimen in ventral view.

*Cryptocephalus moraei* (Linnaeus, 1758) is a small beetle measuring 3 to 4 mm ( Brock, 2021). It belongs to the family Chrysomelidae, commonly referred to as leaf beetles, and species from the *Cryptocephalus* genus are often called pot beetles. The species is shiny black, with strongly punctured striae on the elytra. It has a distinctive pattern that allows for reliable field identification: four small yellow or orange patches, one in the lateral and one in the posterior part of each elytron ( Figure 1). The species is fully winged and capable of flight ( Cox, 2004).

*Cryptocephalus moraei* feeds on the leaves and pollen of St John’s-wort *Hypericum* sp., with a preference for *H. perforatum* L. ( Erber, 1988; James, 2018). The beetle prefers open, rough habitat on chalk or gravel, calcareous grassland and heathland, but can also be found in broad-leaved woodland ( Cox, 1991; James, 2018). Widespread in England and Wales, particularly south of The Wash, but scarcer and more scattered further north; there is a single record from Scotland: North Ayrshire on 27/07/1895 (specimen at the Glasgow Museum) ( Burgess, 2020; Chrysomelidae Recording Scheme, 2023).

This beetle is oviparous. The adults mate in the spring and the female produces a few hundred eggs over several weeks. When ovipositing the female covers the fertilised egg in excrement and deposits it on the ground. There are four larval instars. The newly hatched larvae retain their case and expand it by adding more excrement. When mature, the larva attaches the case to a leaf or bark, closes the opening and pupates inside. *Cryptocephalus moraei* overwinters as a larva ( Cox, 1991; UK Beetles, no date). The adults are active from May to September, peaking in June and July ( UK Beetles, no date).

*Cryptocephalus moraei* has been identified from a Pleistocene fossil from Rodbaston Hall ( Ashworth, 1973; SEAD, no date) and Late Bronze Age material from Runnymede ( Jolivet, 2008; Robinson, 2000; SEAD, no date).

The high-quality genome of *Cryptocephalus moraei* was sequenced as part of the Darwin Tree of Life Project, a collaborative effort to sequence all named eukaryotic species in the Atlantic Archipelago of Britain and Ireland. Here we present a chromosomally complete genome sequence for *Cryptocephalus moraei* based on two specimens from Parsonage Moor and Cothill Fen Nature Reserves, England.

## Genome sequence report

The genome was sequenced from one female *Cryptocephalus moraei* ( Figure 1) collected from Dry Sandford Pit Nature Reserve (51.69, –1.33). A total of 35-fold coverage in Pacific Biosciences single-molecule HiFi long reads was generated. Primary assembly contigs were scaffolded with chromosome conformation Hi-C data. Manual assembly curation corrected 43 missing joins or mis-joins and removed 6 haplotypic duplications, reducing the assembly length by 0.36% and the scaffold number by 9.15%, and decreasing the scaffold N50 by 0.47%.

**Figure 2.**
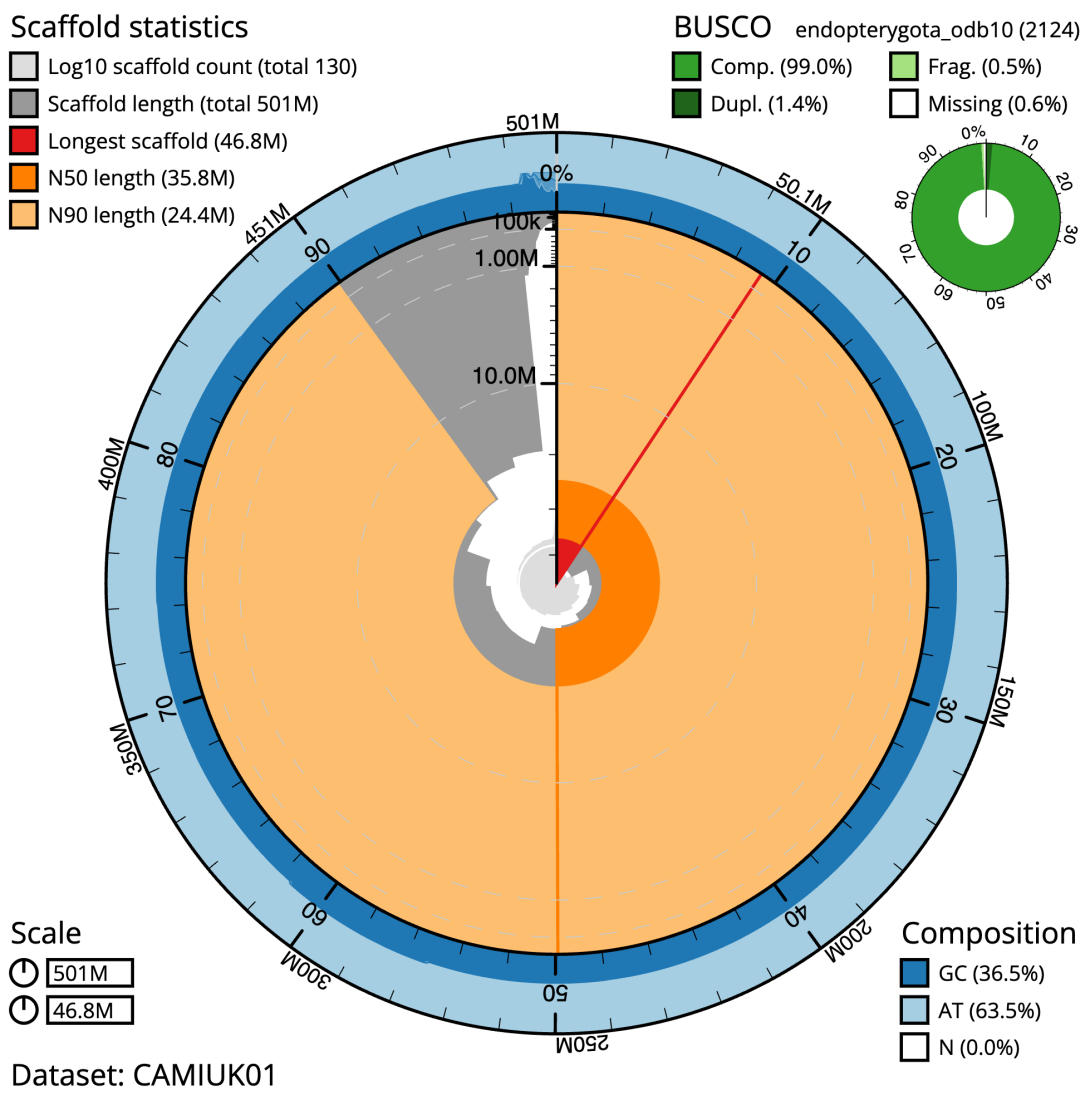
Genome assembly of *Cryptocephalus moraei*, icCryMora2.1: metrics. The BlobToolKit Snailplot shows N50 metrics and BUSCO gene completeness. The main plot is divided into 1,000 size-ordered bins around the circumference with each bin representing 0.1% of the 500,560,161 bp assembly. The distribution of scaffold lengths is shown in dark grey with the plot radius scaled to the longest scaffold present in the assembly (46,835,023 bp, shown in red). Orange and pale-orange arcs show the N50 and N90 scaffold lengths (35,831,563 and 24,393,232 bp), respectively. The pale grey spiral shows the cumulative scaffold count on a log scale with white scale lines showing successive orders of magnitude. The blue and pale-blue area around the outside of the plot shows the distribution of GC, AT and N percentages in the same bins as the inner plot. A summary of complete, fragmented, duplicated and missing BUSCO genes in the endopterygota_odb10 set is shown in the top right. An interactive version of this figure is available at https://blobtoolkit.genomehubs.org/view/icCryMora2.1/dataset/CAMIUK01/snail.

The final assembly has a total length of 500.5 Mb in 129 sequence scaffolds with a scaffold N50 of 35.8 Mb ( Table 1). Most (98.26%) of the assembly sequence was assigned to 15 chromosomal-level scaffolds, representing 14 autosomes and the X sex chromosome. Chromosome-scale scaffolds confirmed by the Hi-C data are named in order of size ( Figure 2– Figure 5; Table 2). The X chromosome was identified using that of the *Phyllotreta cruciferae* genome (GCA_917563865.1) While not fully phased, the assembly deposited is of one haplotype. Contigs corresponding to the second haplotype have also been deposited. The mitochondrial genome was also assembled and can be found as a contig within the multifasta file of the genome submission.

**Table 1.** Genome data for *Cryptocephalus moraei*, icCryMora2.1.

Project accession data		
Assembly identifier	icCryMora2.1	
Species	*Cryptocephalus moraei*	
Specimen	icCryMora2	
NCBI taxonomy ID	204949	
BioProject	PRJEB54797	
BioSample ID	SAMEA11025075	
Isolate information	icCryMora2, female: whole organism (DNA sequencing) icCryMora1, female: whole organism (Hi-C scaffolding)	
Assembly metrics *		*Benchmark*
Consensus quality (QV)	61.3	*≥ 50*
*k*-mer completeness	100%	*≥ 95%*
BUSCO **	C:99.0%[S:97.6%,D:1.4%], F:0.5%,M:0.6%,n:2,124	*C ≥ 95%*
Percentage of assembly mapped to chromosomes	98.26%	*≥ 95%*
Sex chromosomes	X chromosome	*localised homologous pairs*
Organelles	Mitochondrial genome assembled	*complete single alleles*
Raw data accessions		
PacificBiosciences SEQUEL II	ERR9981091	
Hi-C Illumina	ERR9988133	
Genome assembly		
Assembly accession	GCA_946251905.1	
*Accession of alternate haplotype*	GCA_946251935.1	
Span (Mb)	500.5	
Number of contigs	254	
Contig N50 length (Mb)	9.6	
Number of scaffolds	129	
Scaffold N50 length (Mb)	35.8	
Longest scaffold (Mb)	46.8	

* Assembly metric benchmarks are adapted from column VGP-2020 of “Table 1: Proposed standards and metrics for defining genome assembly quality” from ( Rhie *et al.*, 2021). ** BUSCO scores based on the endopterygota_odb10 BUSCO set using v5.3.2. C = complete [S = single copy, D = duplicated], F = fragmented, M = missing, n = number of orthologues in comparison. A full set of BUSCO scores is available at https://blobtoolkit.genomehubs.org/view/icCryMora2.1/dataset/CAMIUK01/busco.

**Figure 3.**
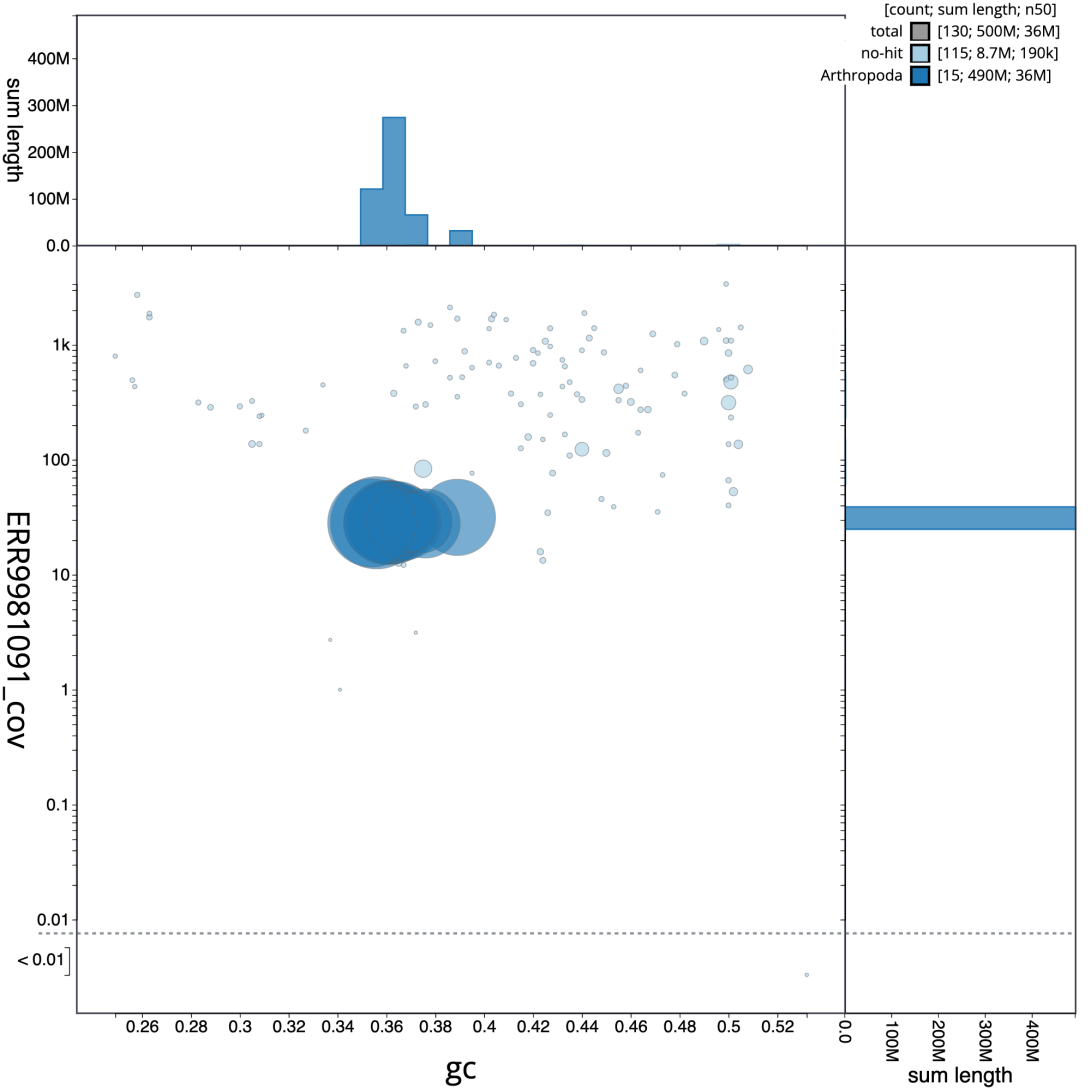
Genome assembly of *Cryptocephalus moraei*, icCryMora2.1: BlobToolKit GC-coverage plot. Scaffolds are coloured by phylum. Circles are sized in proportion to scaffold length. Histograms show the distribution of scaffold length sum along each axis. An interactive version of this figure is available at https://blobtoolkit.genomehubs.org/view/icCryMora2.1/dataset/CAMIUK01/blob.

**Figure 4.**
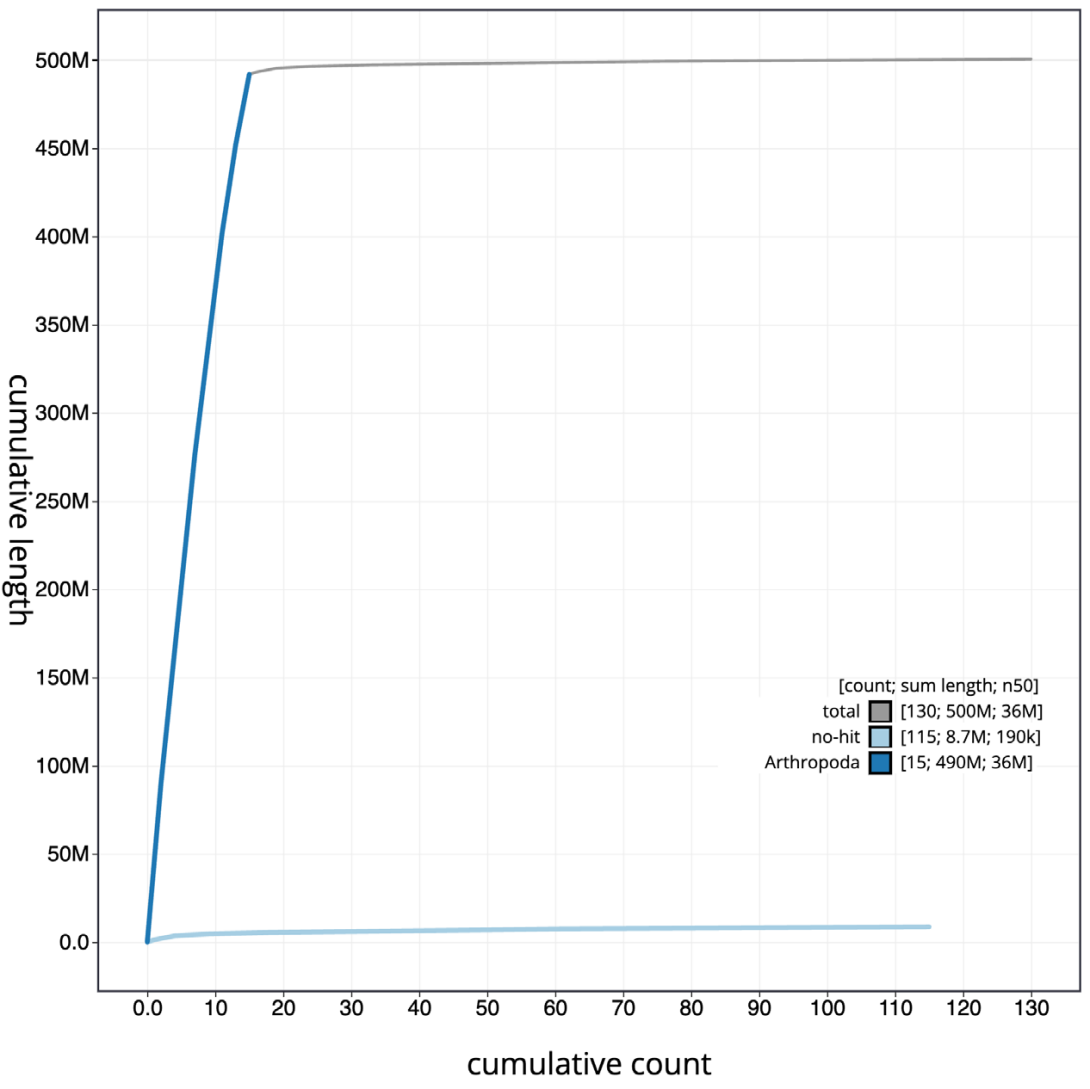
Genome assembly of *Cryptocephalus moraei*, icCryMora2.1: BlobToolKit cumulative sequence plot. The grey line shows cumulative length for all scaffolds. Coloured lines show cumulative lengths of scaffolds assigned to each phylum using the buscogenes taxrule. An interactive version of this figure is available at https://blobtoolkit.genomehubs.org/view/icCryMora2.1/dataset/CAMIUK01/cumulative.

**Figure 5.**
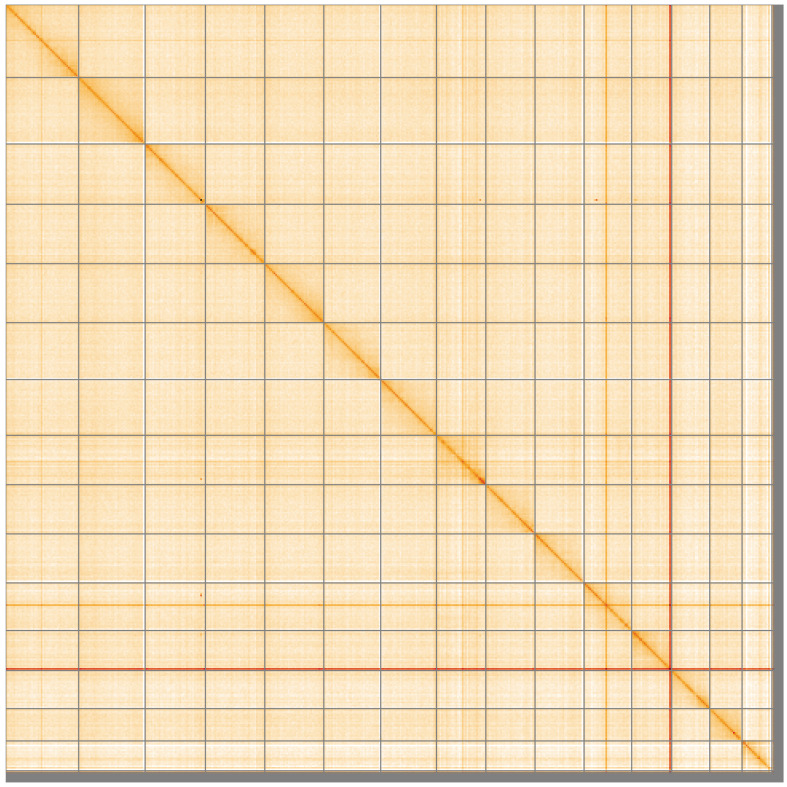
Genome assembly of *Cryptocephalus moraei*, icCryMora2.1: Hi-C contact map of the icCryMora2.1 assembly, visualised using HiGlass. Chromosomes are shown in order of size from left to right and top to bottom. An interactive version of this figure may be viewed at https://genome-note-higlass.tol.sanger.ac.uk/l/?d=AU7k8c4tT3WzJdclLXS5zA.

**Table 2.** Chromosomal pseudomolecules in the genome assembly of *Cryptocephalus moraei*, icCryMora2.

INSDC accession	Chromosome	Length (Mb)	GC%
OX276452.1	1	46.84	35.5
OX276454.1	2	38.71	36.0
OX276455.1	3	38.13	36.0
OX276456.1	4	37.9	36.0
OX276457.1	5	36.47	36.0
OX276458.1	6	35.83	36.5
OX276459.1	7	31.68	39.0
OX276460.1	8	31.62	36.0
OX276461.1	9	31.45	36.0
OX276462.1	10	30.54	36.5
OX276463.1	11	25.74	37.5
OX276464.1	12	24.39	36.5
OX276465.1	13	20.75	37.5
OX276466.1	14	19.15	37.0
OX276453.1	X	42.66	35.5
OX276467.1	MT	0.02	25.0

The estimated Quality Value (QV) of the final assembly is 61.3 with *k*-mer completeness of 100%, and the assembly has a BUSCO v5.3.2 completeness of 99.0% (single = 97.6%, duplicated = 1.4%), using the endopterygota_odb10 reference set ( *n* = 2,124).

Metadata for specimens, spectral estimates, sequencing runs, contaminants and pre-curation assembly statistics can be found at https://links.tol.sanger.ac.uk/species/204949.

## Methods

### Sample acquisition and nucleic acid extraction

A *Cryptocephalus moraei* (NHMUK014036740, icCryMora2) was collected from Dry Sandford Pit Nature Reserve, England (latitude 51.69, longitude –1.33) on 2021-06-19 using an aerial net. The specimen was collected as a Master record as part of the eMesozoic digitisation project by Olga Sivell and Duncan Sivell (Natural History Museum). A second specimen (NHMUK014036774, icCryMora1) was collected on 2021-06-19 by Olga Sivell and Ryan Mitchell at Cothill Fen National Nature Reserve (51.69, –1.33). The specimens were both identified by Duncan Sivell using Hubble (2012). The specimens were preserved on dry ice.

The specimen was prepared for DNA extraction at the Tree of Life laboratory, Wellcome Sanger Institute (WSI). The icCryMora2 sample was weighed and dissected on dry ice. Whole organism tissue was disrupted using a Nippi Powermasher fitted with a BioMasher pestle. DNA was extracted at the Wellcome Sanger Institute (WSI) Scientific Operations core using the Qiagen MagAttract HMW DNA kit, according to the manufacturer’s instructions.

### Sequencing

Pacific Biosciences HiFi circular consensus DNA sequencing libraries were constructed according to the manufacturers’ instructions. DNA sequencing was performed by the Scientific Operations core at the WSI on Pacific Biosciences SEQUEL II (HiFi) instrument. Hi-C data were also generated from whole organism tissue of icCryMora1 using the Arima2 kit and sequenced on the icCryMora2 instrument.

### Genome assembly, curation and evaluation

Assembly was carried out with Hifiasm ( Cheng *et al.*, 2021) and haplotypic duplication was identified and removed with purge_dups ( Guan *et al.*, 2020). The assembly was then scaffolded with Hi-C data ( Rao *et al.*, 2014) using YaHS ( Zhou *et al.*, 2023). The assembly was checked for contamination and corrected as described previously ( Howe *et al.*, 2021). Manual curation was performed using HiGlass ( Kerpedjiev *et al.*, 2018) and Pretext ( Harry, 2022). The mitochondrial genome was assembled using MitoHiFi ( Uliano-Silva *et al.*, 2022), which runs MitoFinder ( Allio *et al.*, 2020) or MITOS ( Bernt *et al.*, 2013) and uses these annotations to select the final mitochondrial contig and to ensure the general quality of the sequence.

A Hi-C map for the final assembly was produced using bwa-mem2 ( Vasimuddin *et al.*, 2019) in the Cooler file format ( Abdennur & Mirny, 2020). To assess the assembly metrics, the *k*-mer completeness and QV consensus quality values were calculated in Merqury ( Rhie *et al.*, 2020). This work was done using Nextflow ( Di Tommaso *et al.*, 2017) DSL2 pipelines “sanger-tol/readmapping” ( Surana *et al.*, 2023a) and “sanger-tol/genomenote” ( Surana *et al.*, 2023b). The genome was analysed within the BlobToolKit environment ( Challis *et al.*, 2020) and BUSCO scores ( Manni *et al.*, 2021; Simão *et al.*, 2015) were calculated.

Table 3 contains a list of relevant software tool versions and sources.

**Table 3.** Software tools: versions and sources.

Software tool	Version	Source
BlobToolKit	4.1.5	https://github.com/blobtoolkit/blobtoolkit
BUSCO	5.3.2	https://gitlab.com/ezlab/busco
Hifiasm	0.16.1-r375	https://github.com/chhylp123/hifiasm
HiGlass	1.11.6	https://github.com/higlass/higlass
Merqury	MerquryFK	https://github.com/thegenemyers/MERQURY.FK
MitoHiFi	2	https://github.com/marcelauliano/MitoHiFi
PretextView	0.2	https://github.com/wtsi-hpag/PretextView
purge_dups	1.2.3	https://github.com/dfguan/purge_dups
sanger-tol/ genomenote	v1.0	https://github.com/sanger-tol/genomenote
sanger-tol/ readmapping	1.1.0	https://github.com/sanger-tol/readmapping/tree/1.1.0
YaHS	yahs- 1.1.91eebc2	https://github.com/c-zhou/yahs

### Legal and ethical review process for Darwin Tree of Life Partner submitted materials

The materials that have contributed to this genome note have been supplied by a Darwin Tree of Life Partner.

The submission of materials by a Darwin Tree of Life Partner is subject to the **‘Darwin Tree of Life Project Sampling Code of Practice’**, which can be found in full on the Darwin Tree of Life website here. By agreeing with and signing up to the Sampling Code of Practice, the Darwin Tree of Life Partner agrees they will meet the legal and ethical requirements and standards set out within this document in respect of all samples acquired for, and supplied to, the Darwin Tree of Life Project.

Further, the Wellcome Sanger Institute employs a process whereby due diligence is carried out proportionate to the nature of the materials themselves, and the circumstances under which they have been/are to be collected and provided for use. The purpose of this is to address and mitigate any potential legal and/or ethical implications of receipt and use of the materials as part of the research project, and to ensure that in doing so we align with best practice wherever possible.

The overarching areas of consideration are:

- Ethical review of provenance and sourcing of the material
- Legality of collection, transfer and use (national and international)

Each transfer of samples is further undertaken according to a Research Collaboration Agreement or Material Transfer Agreement entered into by the Darwin Tree of Life Partner, Genome Research Limited (operating as the Wellcome Sanger Institute), and in some circumstances other Darwin Tree of Life collaborators.

## Data Availability

European Nucleotide Archive: *Cryptocephalus moraei*. Accession number PRJEB54797; https://identifiers.org/ena.embl/PRJEB54797. ( Wellcome Sanger Institute, 2022) The genome sequence is released openly for reuse. The *Cryptocephalus moraei* genome sequencing initiative is part of the Darwin Tree of Life (DToL) project. All raw sequence data and the assembly have been deposited in INSDC databases. The genome will be annotated using available RNA-Seq data and presented through the Ensembl pipeline at the European Bioinformatics Institute. Raw data and assembly accession identifiers are reported in Table 1.

## Author information

Members of the Natural History Museum Genome Acquisition Lab are listed here: https://doi.org/10.5281/zenodo.4790042.

Members of the Darwin Tree of Life Barcoding collective are listed here: https://doi.org/10.5281/zenodo.4893703.

Members of the Wellcome Sanger Institute Tree of Life programme are listed here: https://doi.org/10.5281/zenodo.4783585.

Members of Wellcome Sanger Institute Scientific Operations: DNA Pipelines collective are listed here: https://doi.org/10.5281/zenodo.4790455.

Members of the Tree of Life Core Informatics collective are listed here: https://doi.org/10.5281/zenodo.5013541.

Members of the Darwin Tree of Life Consortium are listed here: https://doi.org/10.5281/zenodo.4783558.
