# Supplementary material for: The genome sequence of a leaf beetle, Cryptocephalus moraei (Linnaeus, 1758)
Source: Wellcome Open Res. 2023 Oct 13;8:467. [Version 1] doi: 10.12688/wellcomeopenres.19522.1 (PMC11053346; doi:10.12688/wellcomeopenres.19522.1)
Supplement: Peer review history

# Reviewer report: Reviewer response for version 1

**DOI:** 10.21956/wellcomeopenres.21625.r76558

**Published:** 2024-04-26

**Authors:** Matteo Montagna, Carmela Gissi

**Affiliations:** 1 Department of Agricultural Sciences, University of Naples Federico II, Portici, Naples, Italy; 2 Universita degli Studi di Bari Aldo Moro, Bari, Apulia, Italy

In the present study, the authors developed a reference genome for the leaf beetle *Cryptocephalus moraei*. This paper clearly reports the results obtained and relevant scientific information is also provided. However, in my view, some aspects can be improved. Below I have reported my comments.

1. Since this paper provides the reference genome of a species, in my view, it is important to give a more comprehensive overview of the ecology/phenology and distribution of the species, not referring only to the UK administrative boundaries. Regarding the distribution, I suggest referring (Staines C, et al., 2011 [Ref 1]). In literature are reported also the following host plants: *Galium lateum*, *Spartum scoparius*, *Scabiosa* spp., *Sarothamnus* spp., *Achillea* spp., *Trifolium* spp., *Ostrya carpinifolia*, *Crataegus monogyna*, *Onobrychis vicifolia*, *Salvia nemorosa*, *Pinus nigra*. If the authors want to add the previous host plants, here are the references: A. Gok, E. G. Cilbiroglu, 2003. The Chrysomelidae fauna of Kovada Stream Arboretum (Egirdir-Isparta, Turkei) (Coleoptera). Nouv. Rev. Ent., 20, 1: 61-73. A. Crişan, 2007. Researches on leaf-beetles (Coleoptera: Chrysomelidae) in the black pine of Banat. Entomol. rom., 11: 13-18. Hardersen S., 2008. Research on the natural heritage of the reserves vincheto di celarda and Val Tovanella (Belluno province, Italy). Sassi D., 2007.  I Crisomelidi del Triangolo Lariano (Italia, Lombardia), Natura. Rivista di Scienze Naturali, 96(2). Gavrilovic B., 2014. Leaf Beetles (Coleoptera: Chrysomelidae) of Mt. Fruška Gora (Vojvodina province, Northern Serbia), with an overview of host plant. Šumarski list,1–2 (2014): 29-41.
2. The fact that some *C. moraei* specimens were discovered in ~10k years old deposits of Rodbaston Hall is interesting, but without insight into the evolutionary context this sentence becomes irrelevant and unlinked with the previous section. If information on the closest species to *C. moraei* is available in the literature, I suggest its integration to improve this short paragraph.
3. Figure 1. I suggest adding the scale bar in each panel. A higher magnification of the specimen from which the DNA was extracted can be nice. Since the habitat of this species was described, the authors can consider the addition of a picture of the *C. moraei *living habitat together with the figure of the specimen.
4. Genome sequence report section. Line 3 - please report the coordinate as done below (latitude 51.69, longitude –1.33)
5. Methods section:

- To facilitate the application of the laboratory protocols and the bioinformatic pipeline for the genome sequencing of closely related species, I think the following data should be included: *i.* the ng/micrograms of HMW-DNA obtained from a single individual. The result of the Femto Pulse analysis on the extracted HMW-DNA should be included in the supplementary material; *ii.* the absorbance value of the extract HMW-DNA is an important parameter that in my view should be provided; *iii.* Did the authors use Megaruptor3 to fragment HMW-DNA in the 15-20kb range? If yes, please report the used volume and concentration; iv. For PacBio sequencing, did the authors use the SMRTbell prep kit 3.0 or 2.0?
- I suggest better stating the procedure used to extract the DNA and report details. For example, the authors stated that the specimen was dissected in dry ice: which parts of the body were removed? I guess the elytra but I think it is more appropriate to clearly state it.
- Unclear sentence: “Hi-C data were also generated from whole organism tissue of icCryMora1 using the Arima2 kit and sequenced on the icCryMora2 instrument.”.
- Did you also test hifiasm assembly with Hic integration mode?
- Which k-mer size was selected?
- Did you perform the genome profiling with Genomescope2?
- Which software did you use for the assembly of the mtDNA? Did you compare the results of multiple software?
- How did you define and validate the number of chromosomes? Was it based on previous cytogenetic data? If you used information from Alegre & Petitpierre 1982, I suggest citing this paper. While, regarding the X chromosome, if data are available and the analyses do not take much time, I suggest using data from more closely related groups such as Cassidinae, Lamprosomatinae or Eumolpine.

6.    Section: Legal and ethical review process for Darwin Tree of Life Partner submitted materials. If the sentence written here is not a standard declaration provided by the journal, in my view the authors have simply to state that all the collected materials have fulfilled all the requirements established by the Darwin Tree of Life Project Sampling Code of Practice, without providing any justification for what is included in it.

Are sufficient details of methods and materials provided to allow replication by others?

Partly

Is the rationale for creating the dataset(s) clearly described?

Yes

Are the datasets clearly presented in a useable and accessible format?

Yes

Are the protocols appropriate and is the work technically sound?

Yes

Reviewer Expertise:

Insect evolution, insect-plant-microbiota interactions, insect symbiosis, Chrysomelidae systematics

We confirm that we have read this submission and believe that we have an appropriate level of expertise to confirm that it is of an acceptable scientific standard.

## References

[1] : Catalogue of Palaearctic Coleoptera, Volume 6. Chrysomeloidea. *Annals of the Entomological Society of America* .2011;104(3) : 10.1603/008.104.0301 10.1603/008.104.0301

[2] : Chrysomeloidea II (Orsodacnidae, Megalopodidae, Chrysomelidae) – Part 1. *Catalogue of Palaearctic Coleoptera: CTHS* .2024;

[3] : Leaf beetles (Coleoptera: Chrysomelidae) of Mt. Fruška Gora (Vojvodina Province, Northern Serbia), with an overview of host plants. *Sumarski List, 2014, 138, 1-2, 29-41 ISSN: 0373-1332 CTHS* .2014;

# Reviewer report: Reviewer response for version 1

**DOI:** 10.21956/wellcomeopenres.21625.r68954

**Published:** 2023-11-03

**Authors:** Huai-Jun Xue

**Affiliations:** 1 Nankai University, Tianjin, China

In the present study, a genome was assembled to the chromosomal level for an individual female of a leaf beetle species *Cryptocephalus moraei* but without annotation for genes and further analysis. In my opinion, the methods adopted in this study are feasible. I don't have no more comments.

 A minor issue:

- “ *Cryptocephalus moraei *feeds on the leaves and pollen of St John’s-wort *Hypericum *sp., with a preference for *H. perforatum *L.” - If *C. moraei* feeds on more than one species of *Hypericum*, should be “ *Hypericum *spp.”

Are sufficient details of methods and materials provided to allow replication by others?

Yes

Is the rationale for creating the dataset(s) clearly described?

Yes

Are the datasets clearly presented in a useable and accessible format?

Yes

Are the protocols appropriate and is the work technically sound?

Yes

Reviewer Expertise:

Evolution, speciation

I confirm that I have read this submission and believe that I have an appropriate level of expertise to confirm that it is of an acceptable scientific standard.
